# Recent Synthesis, Characterization, and Pharmacological Evaluation of Multifunctional Hemorphins Containing Non-Natural Amino Acids with Potential Biological Importance

**DOI:** 10.3390/ph15111425

**Published:** 2022-11-17

**Authors:** Petar Todorov, Stela Georgieva, Jana Tchekalarova

**Affiliations:** 1 Department of Organic Chemistry, University of Chemical Technology and Metallurgy, 1756 Sofia, Bulgaria; 2 Department of Analytical Chemistry, University of Chemical Technology and Metallurgy, 1756 Sofia, Bulgaria; 3 Institute of Neurobiology, Bulgarian Academy of Sciences, 1113 Sofia, Bulgaria

**Keywords:** hemorphins, non-natural amino acids, unusual amino acids, non-proteinogenic amino acids, biological activity, electrochemical behavior of peptides

## Abstract

The endogenous hemorphins are bioactive peptides with activity on opioid receptors. They are extensively studied and summarized in numerous reviews. During the last decade, several research teams have synthesized, characterized, and pharmacologically evaluated synthetic hemorphin analogs containing unusual amino acids, D-amino acids, α-aminophosphonic acids, and their derivatives. The present review summarizes the current studies on short-chain synthetic hemorphin peptide derivates containing non-natural amino acids. This review focuses on the structure–activity relationship analysis, details on specific methods for their characterization, and the advantage of synthetic hemorphin analogs compared to endogenous peptides as potent biologically active compounds with a complex mechanism of action.

## 1. Introduction

The endogenous peptides have biological activity and originate from precursor proteins via enzyme degradation in vesicles. They are released from the cell upon stimulation to function as neurotransmitters, hormones, and some short-chain peptides with unclear functions. Over the last decade, peptides derived from hemoglobin (Hb) have been extensively explored [1]. In the 1980s, endogenous opioid peptides were identified, leading to the isolation and characterization of Hb-active peptides with opioid-like effects [2]. The proteasomes and oligopeptides are enzymes producing hemoglobin (HB)-derived peptides with variable activities. These Hb-derived short-chain peptides consist of 4 to 10 amino acid residues, obtained from 35–38 and 35–39 fragments of β, γ, δ, and ε chains of Hb in humans, called hemorphins [3,4,5,6,7,8,9,10,11,12]. Hemorphins are endogenous peptides with opioid receptor affinity and morphinomimetic properties [13]. Several review articles have been written about hemorphins, including their isolation, purification, and structure–activity analysis [1,14,15]. Some of the structurally related hemorphins function as opioid receptor ligands with an affinity for µ-, δ-, and k-receptors and antinociceptive activity. In the peripheral nervous system, hemorphins affect cardiovascular, digestive, and endocrine functions. Some hemorphins play an essential role in the regulation of blood pressure by suppressing the activity of angiotensin-converting enzyme (ACE) and insulin-regulating aminopeptidase (IRAP).

The first known opioidergic peptide extracted from Hb (ß-chain 35–38) was hemorphin-4 with a possessed amino acid sequence: Tyr-Pro-Trp-Thr. By treating bovine blood with gastrointestinal enzymes with analytical techniques, its structure has been proven [16]. Hemorphin-4 can also be obtained by enzymatic hydrolysis of casein and Hb [17]. Yang et al. (1999) studied the effects of eight opioid tetrapeptides with similar amino acid sequences: endomorphin-1 (Tyr-Pro-Trp-Phe-NH_2_), endomorphin-2 (Tyr-Pro-Phe-Phe-NH_2_), morphiceptin (Tyr-Pro-Phe-Pro-NH_2_), hemorphin-4 (Tyr-Pro-Trp-Thr), Tyr-MIF-1 (Tyr-Pro-Leu-Gly-NH_2_), Tyr-W-MIF-1 (Tyr-Pro-Trp-Gly-NH_2_), TAPS (Tyr-D-Arg-Phe-Sar), and DALDA (Tyr-D-Arg-Phe-Lys-NH_2_), expressed in the rat locus coeruleus neurons whose brain structure is part of the reticular activating system involved in physiological responses to stress and panic [17]. All of these tetrapeptides spontaneously inhibited the tested neurons in the locus coeruleus. Hemorphin-4 has a similar structure and amino acid sequence close to that of endomorphins. Endomorphins have high affinity and selectivity for opioid receptors and are most responsible for the analgesic effects in the central nervous system. These properties of theirs are due to the presence of Pro, which represents a crucial factor for the structural and conformational properties of the ligand [18,19,20]. Proline plays the role of a stereochemical spacer capable of inducing a favorable spatial orientation of aromatic rings, which, in turn, is a crucial factor for ligand recognition and their interaction with the receptors. Therefore, the replacement of natural amino acids with other small non-natural amino acid modifications and their incorporation into opioid-based peptides have been the subject of intense research in recent years. Mollica et al. (2012, 2014, and 2015) performed detailed and valuable research on the use of various non-natural amino acid modifications as building blocks for drug discovery [18,19,20]. Thus, for example, the replacement of native proline with the divalent amino acid cis-4-amino-L-proline (cAmp) combining the conformational rigidity of the ring in opioid peptides can affect the stereochemistry of the entire molecule peptide, thereby leading to a significant increase in μ-opioid affinity and activity and to the correct fit of the peptide to the receptor [18,19,20]. Due to the interesting structural properties of the cAmp residue, its insertion into a peptide backbone can lead to both the usual linear analogs and some structurally interesting cyclical patterns. Conformational flexibility around Pro can be further enhanced by incorporating achiral analogs of Cα,α-disubstituted glycines, 1-aminocyclopentanecarboxylic acid (Ac5c), and 1-aminocyclohexanecarboxylic acid (Ac6c), etc. It should also be mentioned that the non-natural amino acid 2,6-dimethyltyrosine (Dmt) can increase the bioactivity of the peptide molecule [20], and the insertion of a conformationally restricted α-methylene-β-aminopropanoic acids (Map) residue into peptide molecules can lead to an improvement in the permeability of the blood-brain barrier [21].

The endogenous opioid heptapeptide VV-Hemorphin-5, known as valorphin (Val-Val-Tyr-Pro-Trp-Thr-Gln), is a part of the hemorphin family [22,23,24]. It is produced in the body by proteolytic cleavage of the region 33–39 of the β-globin chain of Hb [24,25]. Valorphin belongs to the endogenous opioid receptor agonists with a preference for the μ-opioid receptor, producing analgesia in animals [23,24]. Despite the relatively low affinity of valorphin for opioid receptors, this peptide, as with classical opioid peptides, effectively inhibits tumor cell growth [26,27]. Over the past decade, several common features have identified a family of growth-inhibitory oligopeptides. They all have a substituted N-terminus, which makes them more resistant to aminopeptidases, a very low optimally active dose (typically picomolar amounts administered in vivo and in vitro), and preferences for certain cells and tissues. This group of peptides includes valorphin, which reversibly inhibits cell proliferation, both in tumor and normal cells [27,28].

The decapeptide LVV-hemorphin-7 (Leu-Val-Val-Tyr-Pro-Trp-Thr-Gln-Arg-Phe) is the largest hemorphin found in high abundance in the mammalian nervous system. It is also the most stable and hydrophobic, in contrast to the other hemorphins [29,30,31]. The structure–activity relationship and potential antihypertensive action of LVV-hemorphins, particularly analogs of LVV-hemorphin-7, have been investigated in detail. The mechanisms by which these analogs act in cardiovascular diseases in rats have also been clarified. It is known that a number of cardiovascular changes, including blood pressure, can be activated via the sympathetic nervous system thanks to the amino acid sequence -Arg-Phe at the C-terminus of hemorphins, as well as all derivatives with a free –COOH or –CONH_2_ group at the C-terminus of the molecule [32]. Furthermore, LVV-hemorphin-6 and LVV-hemorphin-7 can produce anxiolytic effects by reducing anxiety in Wistar rats [33]. Recently, Hung et al. reported a positive link between alcohol-induced anti-nociception and the plasma level of LVV-hemorphin-7 [34]. These findings support the idea for further studies and efforts in developing novel analogs of LVV-hemorphin-7as potential analgesics for alcohol-induced anemia.

The Hb-derived bioactive peptides exert a modulatory role on a cannabinoid–opioid system whose mechanism underlies their implication for treating mood disorders and related behavioral changes. At the end of 2019 and the beginning of 2020, a team of scientists showed the first structural study on the binding of LVV-hemorphin-7 to ACE, IRAP, and the µ-opioid (MOR) receptor. The LVV-hemorphin-7 is a unique peptide in mammals and camels due to arginine replacing the amino acids glutamine. The results showed that camel LVV-hemorphin-7 (Leu-Val-Val-Tyr-Pro-Trp-Thr-Arg-Arg-Phe) has more stable and persistent interactions with all three receptors—MOR, ACE, and IRAP—in contrast to non-camel LVV-hemorphin-7 (Leu-Val-Val-Tyr-Pro-Trp-Thr-Gln-Arg-Phe). Further studies at the cellular and molecular level may elucidate the potential of hemorphin analogs as therapeutic agents in memory loss, hypertension, and analgesia [35,36,37].

Recently, a broad number of peptide drugs were produced via recombinant DNA technology to be used for the prevention of diabetes, epilepsy, hypertension, chronic pain, and many others. However, there is no summary of the synthetic analogs of biologically active endogenous peptides containing unnatural amino acids and derivatives. Therefore, the present review focuses primarily on their physicochemical characterization and structure–activity analysis without claiming complete comprehensiveness.

## 2. Chemistry and Biology of Synthetic Hemorphin Analogs Containing Non-Natural Amino Acids

**Figure 1:**
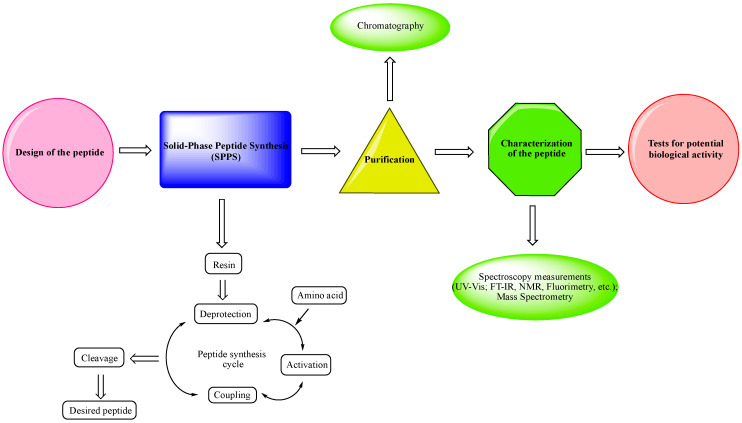
An overview of the peptide pathway—from design to biological testing.

As can be seen in Figure 1, the most schematic pathway from the synthesis of a peptide to its biological tests is:

- Design of the peptide—planning of the desired peptide compound with expected biological activity, what modifications to be made, in which part of the molecule to be made, what properties we expect to obtain, etc.
- Choice of a reliable method used to obtain the desired peptide—peptide synthesis in solution or solid-phase peptide synthesis (SPPS). The solid-phase peptide synthesis by the Fmoc-strategy is the most widespread and acceptable method due to the number of its advantages, including reduced reaction time for creating a peptide bond; quantitative progression of condensation reactions; the easy removal of excess reagents and solvents by washing the peptidyl-resin; minimal losses when receiving the final product.
- The synthesized peptide must be purified using chromatography (the most used is reversed-phase high-performance liquid chromatography (RP-HPLC)).
- Followed by the complete characterization of the peptide using modern instrumental methods and techniques: spectroscopy measurements (UV-Vis; FT-IR, NMR, fluorimetry, etc.); mass spectrometry.
- Screening tests for potential biological activity.

**Figure 2:**
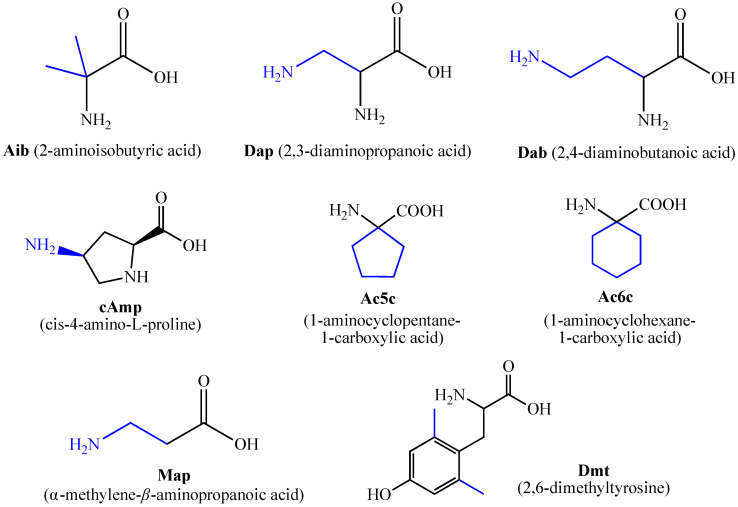
Some of the most used unnatural amino acids.

Changes occurring in the amino acid scaffold can lead to the preparation of new biologically active molecules with potential application in drug design and medicinal chemistry [20,32,38,39,40]. Some of the most usually used non-natural and non-proteinogenic amino acids that are introduced into peptide chains are shown in Figure 2:

Natural amino acids are replaced by non-natural and unusual amino acids, D-amino acids, α-aminophosphonic acids, and their derivatives with very different purposes: obtaining a desired conformation of the peptide; obtaining the desired biological activity; increasing their resistance to enzymatic degradation; improving the stability, efficacy, bioavailability, and other essential properties of the peptides. Therefore, the proper manipulation of amino acid residues in the peptide chain, if successful, would significantly impact the future application of synthetic peptides with non-natural amino acids [32,39,40,41,42,43].

In Table 1, the most active peptide hemorphin analogs, synthesized by our research team over the last five years, are shown.

**Figure 3:**
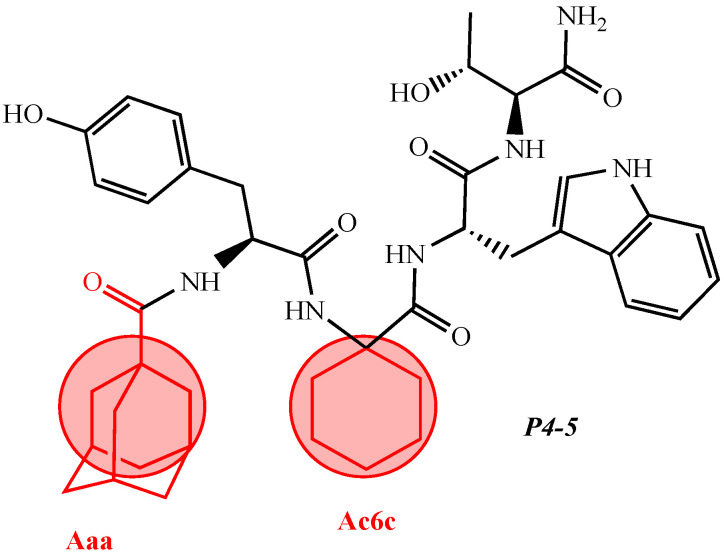
Chemical structure of peptide analog P4-5.

The structure–activity relationship of a series of new analogs of the shortest of all hemorphins, hemorphin-4, was elucidated by Todorov et al. (compounds P4-1, P4-2, P4-3, P4-4, and P4-5) [44]. Modifications have been made by replacing Pro at position 2 with the unnatural and conformationally restricted amino acids Ac5c and Ac6c, as well as introducing the adamantane residue from the N-terminus of the hemorphin-4 molecule that would lead to multiple target systems. The most potent anticonvulsant activity has been exhibited by the peptide analog P4-5, which contains an adamantane residue at the N-terminus and a cyclohexane ring at position 2 (Figure 3). The peptide analog P4-5 had the lowest ED_50_ among other hemorphin-4 peptide analogs and the protective index (PI) with an appropriate safety margin in the MES test (Table 2) that proves the efficacy of active agents against partial and generalized seizure-type epilepsy [56]. In addition, the P4-5 analog exhibited higher potency in the MES test than in the referent angiotensin (Ang) IV [44]. Furthermore, the structure–activity relationship analysis suggests that the presence of an adamantane residue at the N-terminus seems crucial for the anticonvulsant activity in the MES test, and, therefore, against the seizure spread. Interestingly, the expressed biological activity of P4-5 is likely due to an appropriate conformational fit of the peptide to the insulin-regulated aminopeptidase (IRAP) receptor and the great lipophilicity and hydrophobicity of the molecule [44]. It is known that hemorphins and Ang IV could bind to the receptor system (IRAP) and share a common metabolic pathway [30]. Recently, it has been reported that both P4-4 and P4-5 peptide analogs are positively charged based on their pKa constants and the isoelectric points at pH 7.4 [44]. In the 6 Hz test, considered a model of drug-resistant epilepsy [57], the P4-5 peptide analog exerted an effect comparable to those of the other two peptide analogs as follows: P4-1: ED_50_ = 0.52; P 4-4: ED_50_ = 0.44; P4-5: ED_50_ = 0.64 mg/kg (Table 3). It is suggested that sodium channels are the targets of drugs with activity against 6 Hz psychomotor seizures [58]. The P4-4 and P4-5 peptide analogs demonstrated the lowest seizure threshold for intravenous pentylenetetrazol (ivPTZ)-induced clonic seizures (Table 4). It is suggested that drugs with potency against the clonic phase in this test might affect the GABAA receptor complex and GABA-ergic neurotransmission [59].

The Tyr-Pro-Trp fragment is involved in the modulation of numerous biological processes through the activation of opioid receptors [22] and, thus, participates in [38,60]. In addition, the adamantane and cycloalkyl groups provide the desired membrane permeability and conformational fit in the intercellular space enabling efficient transport across lipid membranes [58,61]. Adamantane-based compounds are widely used in practice as potential agents for treating neurological and antiviral conditions, malaria, type 2 diabetes, and inflammatory conditions [62,63]. Some modified adamantane compounds have been reported to show anticonvulsant activity in animal models. In addition to its anticonvulsant properties, the adamantane residue can also act as an analgesic in mouse models [64,65].

In the last year, Todorov’s group [47,52] showed the promising antiviral and antibacterial activity of some new N- and C-modified hemorphin analogs containing different amino acids (Cys, Glu, and His), 1-adamantane carboxylic acid, and niacin against the human respiratory syncytial virus (HRSV-S2) and human adenovirus serotype 5 (HAdV-5) and against B. cereus and P. Aeruginosa (compounds C-V, H-V, AC-V, AH-V NH7C, and NCH7) [47]. The authors were the first to investigate the structural-textile application and potential antimicrobial activities of both hemorphin derivatives and hemorphin-treated textile material [47,52].

The insertion of chromophoric groups that possess interesting features into peptides for photodynamic control of peptide biomolecules has been investigated intensively in recent years [66,67,68]. The influence of cis(Z)- and trans(E)- isomers of recently synthesized biopeptide-bearing azobenzene on the N-side chain of hemorphin-4 has been studied (compound AzP) [46]. Moreover, some researchers have synthesized, characterized, and investigated the structure-related properties of new rhodamineB-conjugated hemorphin-4 analogs as potential sensitive fluorescent probes (compounds № 9–11). These hybrid peptides contain different aliphatic amino acid residues between the chromophoric group, rhodamine B to the N-side, and the amino acid scaffold of natural hemorphin-4 [47].

**Figure 4:**
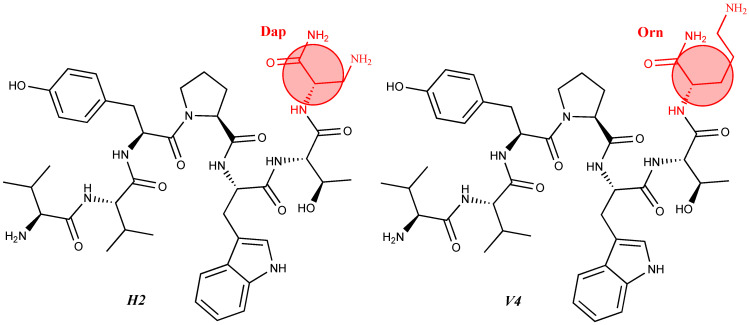
Chemical structure of H2 and V4 peptide analogs.

The idea of introducing non-proteinogenic and natural amino acids for the synthesis of new analogs of VV-hemorphin-5 modified from the C- and N-termini (compounds № 12–17) has been successfully carried out by Todorov et al. [48,49], obtaining peptide structures with the sequences: Xxx-Val-Val-Tyr-Pro-Trp-Thr-Gln-NH_2_ and Val-Val-Tyr-Pro-Trp-Thr-Yyy-NH_2_, where Xxx is Ile or Aib (α-aminoisobutyric acid) and Yyy are Lys/Orn/Dap (2,3-diaminopropanoic acid)/Dab (2,4-diaminobutanoic acid) (see Figure 4). All of these new peptide molecules have been tested for anticonvulsant and potential antinociceptive activities in mice, with the derivative H2 (Val-Val-Tyr-Pro-Trp-Thr-Dap-NH_2_) showing the highest biological activity, in whose structure glutamine is replaced with Dap. In comparison, the derivative V4 (Val-Val-Tyr-Pro-Trp-Thr-Orn-NH_2_), containing a non-proteinogenic amino acid Orn at the C-terminal, showed pronounced anticonvulsant activity, comparable to that of natural valorphin (Table 2, Table 3 and Table 4) [48,49]. None of the newly synthesized analogs of VV-Hemorphin-5 affected motor coordination. While V4 and V5 analogs had similar ED values in the MES test (V4: ED_50_ = 3.63 and V5: ED_50_ = 3.19), V4 exhibited an activity comparable to that of V6 against the 6 Hz psychomotor seizures (V4: ED_50_ = 5.09 and V6: ED_50_ = 5.55) (Table 2 and Table 3). The in silico analysis suggested that changes in Position 7 (replacement of Gln by Lys) must be the crucial factor responsible for the anticonvulsant activity of V5 against generalized seizures in the MES test and activated opioid δ receptors [67]. On the other hand, this activation might be associated with the insertion of Ile at Position 1 in the V6 activity against psychomotor seizures.

Moreover, the V4 peptide increased the threshold for clonic seizures induced by ivPTZ in the lowest dose of 5 µg, comparable to the positive control. The universal potency demonstrated by V4 in three seizure tests with a different mechanism of action might be due to the insertion of amino acid Orn at Position 7 of VV-5 predisposed to various targets. Therefore, the position of replacement and the nature of the inserted group in recently synthesized VV-Hemorphin-5 analogs containing nonproteinogenic and natural amino acids seem critical factors in determining the anticonvulsant and antinociceptive activity of the associated receptor binding.

An active valorphin analog was obtained as a potent inhibitor of dipeptidyl peptidase III by intermolecular C–H arylation on the resin between Trp at position 5 and Tyr at position 3 by using solid-phase peptide synthesis. This peptide is structurally close to spinorphin (Leu-Val-Val-Tyr-Pro-Trp-Thr), an endogenous peptide with antinociceptive action [69,70,71].

For the first time, α-aminophosphonic acids have been introduced into hemorphin peptides (compounds № 18–22 and 41–44) [50,51]. α-Aminophosphonates and aminophosphonic acids occupy an essential place among compounds containing a P-C bond and an amino group. They are structural analogs of natural α-amino acids, which are the “building blocks” of peptides and proteins. Their structure is of interest due to their diverse biological role. The obtained N-modified analogs of VV-hemorphin-5 containing an aminophosphonic residue have been described in detail in terms of structure–activity and have been investigated for antinociceptive and anticonvulsant activity. In the literature, it has been reported that the most potent hemorphin derivative was the V3p, with the lowest ED_50_ of 4.31 µg against psychomotor seizures and ivPTZ clonic seizures (Table 3 and Table 4) [50]. The results of the docking study of the obtained in vivo results suggest that binding to the k-opioid receptor is the most likely mechanism of action of the peptide derivatives with anticonvulsant activity. These data lead us to hypothesize that modification of the two N-terminal Val in the peptide molecules with an aminophosphonate residue in phosphopeptide analogs leads to significant changes in peptide activity and affinity [50,51].

For the first time, C-5-substituted hydantoins were introduced into hemorphins, aiming for a synergistic effect to enhance anticonvulsant activity (compounds № 6, 7, 23, 24, 45, and 46) [45]. Of these hybrid structures, the strongest anticonvulsant activity was reported for VV-hemorphin-5, possessing a 5,5′-diphenylhydantoin residue at the N-terminus and a hydrophobic Val–Val–Tyr–Pro–Trp–Thr–Gln–CONH_2_ amino acid sequence of the peptide molecule (Ph5). This compound showed low ED_50_ for MES and the 6 Hz test, respectively, compared to other tested peptide analogs (Table 2 and Table 3). In silico analysis suggests that the underlying mechanism of the anticonvulsant effect of Ph-5 involves blocking sodium channels [45].

A series of Phe-modified analogs of hemorphin-7-NH_2_ were synthesized and characterized by replacing Phe at position 7 with various natural and unnatural amino acids: Leu, MePhe, D-Phe, Tic, Trp, Met, Oic, Phg (phenylglycine), pNO2Phe, Nle (norleucine), pClPhe, Thi, and Cha. Of all synthetic analogs, the most active are those containing unnatural amino acids: tetrahydro-isoquinoline-3-carboxylic acid (Tic), pClPhe, 3-thienylalanine (Thi), octahydroindole-2-carboxylic acid (Oic), and 3-cyclohexylalanine (Cha). Using phenytoin (5,5′-diphenylhydantoin) as a sodium channel blocker, it has been hypothesized that LVV-hemorphin-7 analogs activate the sympathetic nervous system via interaction with specific receptors functionally linked to phenytoin-sensitive sodium channels. Substitution of Arg at position 6 with Lys slightly reduced blood pressure, in contrast to its substitution with the amino acids citrulline, D-Arg, NO2Arg, Orn, or Ala, where it was significant [28]. Conversion of the C-terminal –COOH group with its amide –CONH_2_ in this type of compound significantly increased the activity of the corresponding peptide analog, indicating that the C-terminal –COOH group is not essential for activity. One possible reason for this is that such a change in the molecule leads to an increase in the resistance of the peptide to enzymatic degradation by endogenous carboxypeptidases. [28,36,37]. Using proteomic studies, the biological role of LVV- and VV-hemorphin-7 as potential biomarkers in patients with posterior cranial fossa brain tumors has been demonstrated. It has been found that the presence of these two hemorphins can be used in the clinical diagnosis of this disease. In the presence of a brain tumor, both hemorphins are not detected in cerebrospinal fluid (CSF) analysis. At the same time, in the case of postoperative removal, they are present [72,73]. 

Two new N- and C-modified analogs of VV-hemorphin-7 containing RGD (Arg–Gly–Asp) residues as potential nociceptive agents and bioactive materials have been elucidated in detail (compounds № 47 and 48) [55]. From the eight LVV- and VV-hemorphin-7 analogs (compounds № 37–44), the H7-1 peptide analog showed the highest potency against the 6 Hz psychomotor seizures with ED_50_ of 0.33 µg (Table 3). However, while the H7-6 had the lowest ED_50_ in the MES test (Table 4), the H7-5 peptide analog raised the ivPTZ-induced clonic seizure at the highest rate at the doses used among the eight synthetized LVV- and VV-hemorphin-7 analogs (Table 4) [54]. Therefore, the modification at the N- and C-terminus with certain amino acids seems to play a critical role in the design of new LVV- and VVhemorphin-7 analogs.

Todorov et al. have synthesized and characterized a series of new analogs of VV-hemorphin-7 (compounds № 29–36) with potential anticonvulsant activity, modified with unnatural amino acids, following the structure Val-Val-Tyr-Xxx-Trp-Thr-Yyy-Arg-Phe-NH_2_, where Xxx is Ac5c (1-aminocyclopentane carboxylic acid) or Ac6c (1-aminocyclohexanecarboxylic acid) and Yyy is Dap (2,3-diaminopropane acid) or Dab (2,4-diaminobutanoic acid) [53]. The peptide analog VV-H5, containing diaminobutanoic acid in its molecule, showed the highest anticonvulsant activity. Moreover, this peptide analog had the lowest ED_50_ of 0.89 µg against psychomotor seizures and ED_50_ of 0.38 µg against the MES among the eight novel compounds (Table 2 and Table 3). In addition, this peptide analog increased the threshold for ivPTZ clonic seizures at the lowest dose of 5 µg injected (Table 4). Interestingly, VV-H5 differs from VV-H4 by only one -CH_2_ group in the molecule, which is crucial for the anticonvulsant activity of this hemorphin derivative.

Table 5 summarizes the different bioactive hemorphin peptides, the test used, concentration/dose, and their potential effects: endogenous tetrapeptides [17]; VV-hemorphin-5 [23,24,25,26,27]; hemorphin-6 and hemorphin-7 [29,32,34,74,75,76,77].

### 2.1. Analytical Characteristics of Hemorphin Analogs

**Figure 5:**
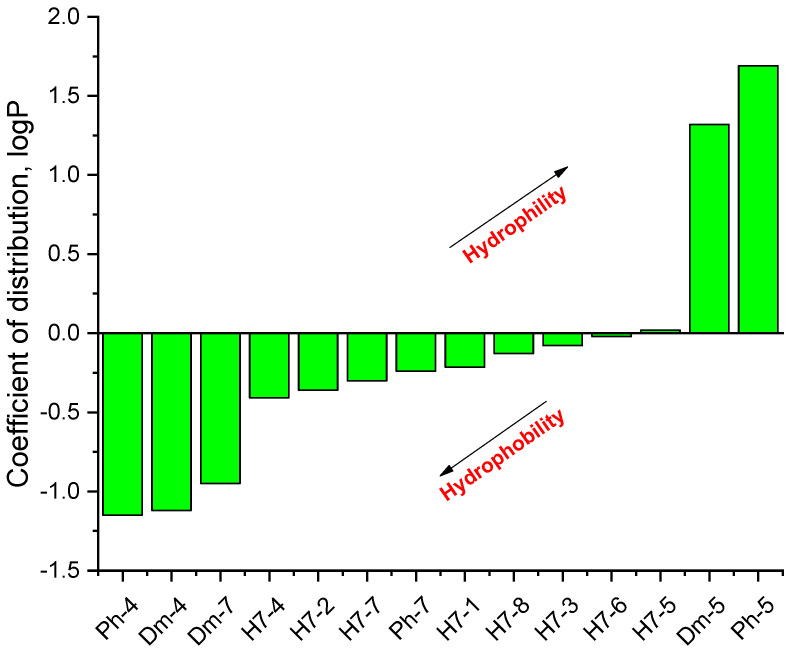
Logarithmic values of the partition coefficient (log P) of some of the investigated hemorphin compounds.

**Figure 6:**
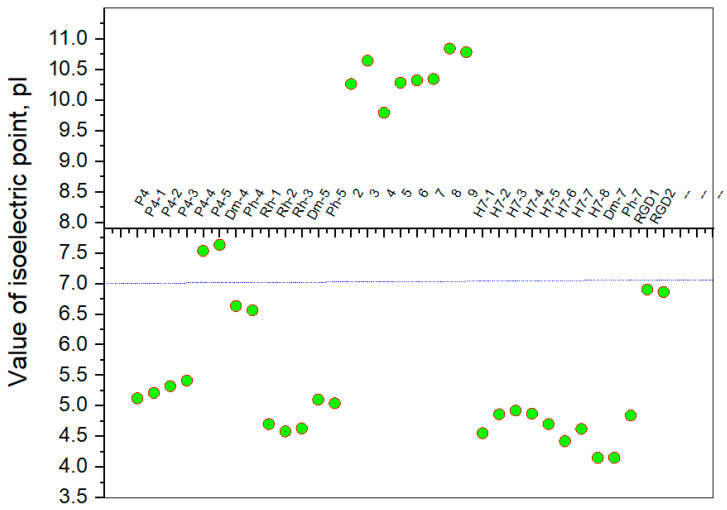
Summary plot of isoelectric point (pI) values of hemorphin analogs.

The structural basis of the biological activity of peptides, i.e., the chemical basis of the extraordinary reactivity of individual amino acid side chains, is one of the most attractive problems in peptide chemistry. It is known that some identical amino acid residues can have different reactivity with respect to given chemical reagents. For example, in an enzyme molecule, only one or a small number of side chains of amino acid residues located in the “active” center can bind substrates or coenzymes, while others with the same chemical composition cannot. As is known, a large part of the hydrophobic side chains is located in the interior of the molecule, thus building a compact core. At the same time, the polar and electron-charged groups are supported on the surface of this matrix. Moreover, the physical and chemical properties of the functional groups are strongly influenced by the nature of the microenvironment. With the same reagent, the same residues but with a different microenvironment exhibit different reactivity. Other important factors for the different reactivity are hydrogen bonds, electrostatic interaction, and steric offenses. The presence of a positive charge, for example, in the vicinity of a given ionizable group, the phenolic group of tyrosine, can facilitate the formation of a labile protonated form mainly in two ways: either by stabilizing the electron pair or by forming another positively charged group. To evaluate the microclimate and behavior of the hemorphin analogs in solution, the distribution coefficient and isoelectric points were determined. Peptides exhibit partial solubility in aqueous (phosphate buffer, pH 6.86 ± 0.01) and (organic) environments, with varying degrees of hydrophilicity and hydrophobicity (Figure 5). Ph-4 and Dm-4 show the greatest hydrophobicity, and Dm-5 and Ph-5 show the greatest tendency to dissolve in organic media. This is due to the fact that the attachment of a non-water-soluble hydantoin component to the main short-chain peptide scaffold stabilizes the zwitterionic form in solutions with a pH of about 7 and interferes with solubility in aqueous solutions. For these compounds, the isoelectric points are around 7.0. pI values close to and around 7 are observed for most short-chain peptide modifications (Figure 6). Compounds P4-4 and P4-5 with modifications Ac5c and Ac6c have pI values around 7, and it is the zwitterionic form in which they will be at this pH that will interfere with their solubility when preparing, for example, injection solutions for biological analyses. Peptide forms with these modifications also showed the least pronounced biological activities. An important parameter evaluating the behavior of non-peptides in solution is also acid–base constants. Determination of the equilibrium constants (pK) of proton dissociation from ionizable side chains of amino acids represents a very important application of spectroscopy and electrochemistry in peptide/protein chemistry. This definition allows conclusions to be drawn regarding the location of these groups in the peptide matrix, as well as their involvement in various interactions. As mentioned, the amino acid fragment of the hemorphin molecule: Tyr-Pro-Trp, is the main sequence thanks to which receptor binding takes place. On the other hand, the amino acids tyrosine and tryptophan, bonded in a peptide chain, are one of the main amino acids exhibiting fluorescent, electrochemical, and acid–basic properties. Table 6 gives the determined pK values of the hemorphin peptides, calculated by applying different analytical techniques, most often by potentiometric titration or mathematical processing of data from the fluorescence/voltammetric analysis. Typically, dissociation of the hemorphin peptide causes changes in the spectrum of one or more chromophores. As the changes are usually small, the spectra of the hemorphin peptide in the ionized form in solution are compared with the same, but in the non-ionized state. At a given pH, part of the total number of chromophores are in the ionized state (α) and has absorption εi, and the rest are in the non-ionized state (1-α) and has absorption ε. Then, the apparent dissociation constant K can be calculated from the Henderson–Hasselball equation: pK = pH-lg[α/(1-α)]. Hemorphin derivatives have acidic properties, which turn them into protolytes of different strengths depending on the amino acid radicals: the more acidic the amino acids in the peptide are, the stronger its acidic properties are expressed. Regardless of the peptide modifications made, the determined acidity constants refer to the side P-groups of tyrosine, the indole nucleus of tryptophan, and arginine in the arginine-containing peptides, exhibiting different degrees of polarity at a pH close to the physiological values of 6–8 (corresponding to the conditions of the cell cytosol). The composition, as well as the polarity, of the P-groups is important for the behavior of the hemorphin peptides in solution, for defining the native conformation, and for the chemical reactions in which they would participate. In the titration curves, during the potentiometric determination of the constants of the peptides with easily ionizable groups, additional sections were obtained, with data from which the pK values were determined. Most peptide derivatives showed approximate pK values related to proton exchange with the -OH group of tyrosine and the indole moiety of tryptophan. As can be seen, peptide derivatives containing a phosphonic group adjacent to the amino acid tyrosine (V2p-V3p series) have weaker protolytic properties, and the protolytic power of long-chain hemorphin derivatives increases with the distance of the -OH group of tyrosine from the corresponding structural modification.

### 2.2. Electrochemical Behavior of Hemorphin Analogs

A comparative review of the electrochemical properties of peptide derivatives would be of interest from a scientific point of view, as the method elucidates fundamental mechanisms of action and the detection of molecular/structural modifications (aggregation). The various biochemical processes such as the functioning of proteins, photosynthesis, and the respiratory chain actively participate in the transfer of protons (PCET—proton-coupled electron transfer) [78,79]. Electrochemical methods add a fresh, new perspective to the research field of hemorphin analogs in terms of their rapid detection, characterization, the study of redox behavior, and electrode response nature. Oxidation of peptides and proteins in aqueous media occurs only at five electroactive amino acid residues, tyrosine [80,81,82], tryptophan [83], histidine, [84], methionine [85], and cysteine [85]. Most of the studied hemorphin derivatives contain only the voltammetrically active tyrosine and tryptophan, connected in the sequence -Tyr-Pro-Trp-, and their oxidation occurs thanks to the presence of p-electrons at the hydroxyl group of the phenol part (Tyr) and indole ring (Trp), respectively [48,49,50]. Studies were carried out in different electrolyte environments by using differently charged surfaces (Table 6). On a glass carbon electrode, at pH~7, the oxidation of hemorphin analogs is irreversible, which occurs at positive potentials (Ep~+0.4 V for Vp series and Ep~+0.7 for H7 series V vs. Ag/AgCl), corresponding to the signal of tyrosine due to the –OH group of the phenol part, which is oxidized to a glass carbonic electrode at a potential close to +0.7 [86]. The oxidation of histidine and cysteine to peptides containing them at a glassy carbon electrode is an irreversible, diffusion-controlled, and pH-dependent process (Table 7) that occurs at Ep~1.2 V vs. Ag/AgCl [81]. A medium of tetrabutylammonium persulfate with methanol proved to be favorable for detecting and determining hemorphin analogs at mercury electrodes, with the resulting well-shaped reduction/oxidation peaks indicating reversible to quasi-reversible electrode processes. Analyses showed that the reactivity of the tyrosine and tryptophan regions was conserved. This, together with the fact that the concentration dependence of the current signal is proportional, gives us a reason to conclude that, in these environments, the structure of the compounds is preserved and aggregation does not occur regardless of the modification of the molecule. Electrochemical methods of investigating the aggregation and fibrillization of hemorphin peptides could become important additional tools in biochemical research, leading to new insights into the molecular mechanisms underlying hemorphin pathogenesis [87,88]. In this regard, an important conclusion can be drawn from the fact that on glassy carbon electrodes, with an increase in the peptide concentration, the current peak of the electroactive amino acid regions decreases/disappears, which refers to a change in the peptide form, most often referring to aggregation of the molecule, which makes electron transfer difficult.

## 3. Conclusions

Our review showed that more than 50 new hemorphin analogs obtained by solid-phase peptide synthesis by the Fmoc-strategy have been synthesized and characterized up to date. These hemorphin peptides contain various unnatural and unusual amino acids, D-amino acids, α-aminophosphonic acids, and their derivatives that are mentioned in this article. Structure–activity relationship studies show that not only the position of the modification, but also the nature of the amino acid involved leads to significant changes in the physicochemical properties (change in pKa, pI, and logP of the peptide), biological activity, and receptor affinity. It can be summarized that even the smallest change in the hemorphin molecule has a great influence on the biological activity, such as the introduction of the unnatural amino acid Dap, and the replacement of Pro with the conformationally constrained amino acids (Ac5c, Ac6c, and adamantane moieties). Structure–activity analysis revealed that the incorporation of an adamantane residue at the N-terminus is necessary for protection against the spread of seizures. Data obtained so far have shown that modification of the two N-terminal Vals in peptide molecules by an aminophosphonate residue in phosphopeptide analogs requires increased biological activity and receptor affinity, from which it can be concluded that the successful design of new analogs of LVV- and VV-hemorphin-7 involves modification of the N- and C-termini with specific amino acids. Predictions from the docking analysis suggest that binding to the k-opioid receptor is the most relevant mechanism of action for the new peptide analogs that possess unnatural amino acids. All hemorphin analogs can be successfully detected at low concentrations by applying voltammetric techniques on solid and mercury electrodes. Electrochemical methods are a good alternative for proving molecular conformational changes and processes of aggregation and peptide fibrillization and can be applied in studying the properties of hemorphin peptides in environments with different matrix compositions.

## Figures and Tables

**Table 1:** Newly synthesized synthetic peptide hemorphin analogs.

№	Abbreviations Given in Articles	Peptide	Molecular Formula	Biological Activity, Reference
		**Hemorphin-4 analogs**		
1	P4-1	Tyr-**Ac5c**-Trp-Thr-NH_2_	C_30_H_38_N_6_O_6_	anticonvulsant activity, [44]
2	P4-2	Tyr-**Ac6c**-Trp-Thr-NH_2_	C_31_H_40_N_6_O_6_	anticonvulsant activity, [44]
3	P4-3	**Aaa**-Tyr-Pro-Trp-Thr-NH_2_	C_40_H_50_N_6_O_7_	anticonvulsant activity, [44]
4	P4-4	**Aaa**-Tyr-**Ac5c**-Trp-Thr-NH_2_	C_41_H_52_N_6_O_7_	anticonvulsant activity, [44]
5	P4-5	**Aaa**-Tyr-**Ac6c**-Trp-Thr-NH_2_	C_42_H_54_N_6_O_7_	anticonvulsant activity, [44]
6	Dm-4	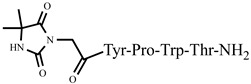	C_36_H_44_N_8_O_9_	anticonvulsant activity, [45]
7	Ph-4	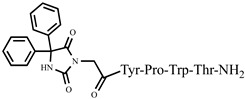	C_46_H_48_N_8_O_9_	anticonvulsant activity, [45]
8	Az-H4	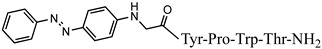	C_43_H_47_N_9_O_7_	anticonvulsant activity, [46]
9	Rh-1	**rhodamineB-Gly**-Tyr-Pro-Trp-Thr-NH_2_	C_59_H_69_N_9_O_9_	antiviral activity, [47]
10	Rh-2	**rhodamineB-β-Ala**-Tyr-Pro-Trp- Thr-NH_2_	C_60_H_71_N_9_O_9_	antiviral activity, [47]
11	Rh-3	**rhodamineB-γ-Abu**-Tyr-Pro-Trp-Thr-NH_2_	C_61_H_73_N_9_O_9_	antiviral activity, [47]
		**Hemorphin-5 analogs**		
12	V2/H2	Val-Val-Tyr-Pro-Trp-Thr-**Dap**-NH_2_	C_42_H_60_N_10_O_9_	antinociceptive and anticonvulsant activity, [48,49]
13	V3/H3	Val-Val-Tyr-Pro-Trp-Thr-**Dab**-NH_2_	C_43_H_62_N_10_O_9_	antinociceptive and anticonvulsant activity, [48,49]
14	V4/H4	Val-Val-Tyr-Pro-Trp-Thr-**Orn**-NH_2_	C_44_H_64_N_10_O_9_	antinociceptive and anticonvulsant activity, [48,49]
15	V5/H5	Val-Val-Tyr-Pro-Trp-Thr-**Lys**-NH_2_	C_45_H_66_N_10_O_9_	antinociceptive and anticonvulsant activity, [48,49]
16	V6/H6	**Ile**-Val-Val-Tyr-Pro-Trp-Thr-Gln-NH_2_	C_50_H_73_N_11_O_11_	antinociceptive and anticonvulsant activity, [48,49]
17	V7/H7	**Aib**-Val-Val-Tyr-Pro-Trp-Thr-Gln-NH_2_	C_48_H_69_N_11_O_11_	antinociceptive and anticonvulsant activity, [48,49]
18	V2p	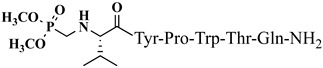	C_42_H_60_N_9_O_12_P	antinociceptive and anticonvulsant activity, [50,51]
19	V3p	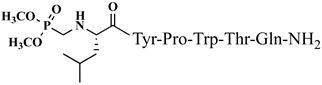	C_43_H_62_N_9_O_12_P	antinociceptive and anticonvulsant activity, [50,51]
20	V4p	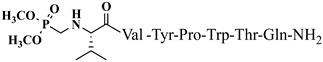	C_47_H_69_N_10_O_13_P	antinociceptive and anticonvulsant activity, [50,51]
21	V5p	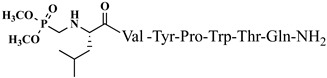	C_48_H_71_N_10_O_13_P	antinociceptive and anticonvulsant activity, [50,51]
22	V6p	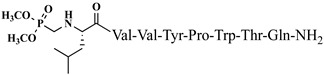	C_53_H_80_N_11_O_14_P	antinociceptive and anticonvulsant activity, [50,51]
23	Dm-5	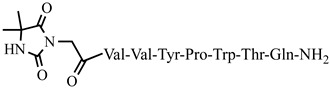	C_51_H_70_N_12_O_13_	anticonvulsant activity, [45]
24	Ph-5	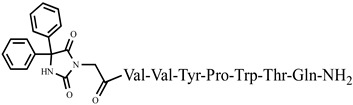	C_61_H_74_N_12_O_13_	anticonvulsant activity, [45]
25	C-V	**Cys**-Val-Val-Tyr-Pro-Trp-Thr-Glu-NH_2_	C_47_H_66_N_10_O_12_S	antiviral and antibacterial activity, [52]
26	H-V	**His**-Val-Val-Tyr-Pro-Trp-Thr-Glu-NH_2_	C_50_H_68_N_12_O_12_	antiviral and antibacterial activity, [52]
27	AC-V	**Aaa-Cys**-Val-Val-Tyr-Pro-Trp-Thr-Glu-NH_2_	C_58_H_80_N_10_O_13_S	antiviral and antibacterial activity, [52]
28	AH-V	**Aaa-His**-Val-Val-Tyr-Pro-Trp-Thr-Glu-NH_2_	C_61_H_82_N_12_O_13_	antiviral and antibacterial activity, [52]
		**Hemorphin-7 analogs**		
29	2	Val-Val-Tyr-**Ac5c**-Trp-Thr-Gln-Arg-Phe-NH_2_	C_60_H_85_N_15_O_12_	anticonvulsant activity, [53]
30	3	Val-Val-Tyr-**Ac6c**-Trp-Thr-Gln-Arg-Phe-NH_2_	C_61_H_87_N_15_O_12_	anticonvulsant activity, [53]
31	4	Val-Val-Tyr-Pro-Trp-Thr-**Dap**-Arg-Phe-NH_2_	C_57_H_81_N_15_O_11_	anticonvulsant activity, [53]
32	5	Val-Val-Tyr-Pro-Trp-Thr-**Dab**-Arg-Phe-NH_2_	C_58_H_83_N_15_O_11_	anticonvulsant activity, [53]
33	6	Val-Val-Tyr-**Ac5c**-Trp-Thr-**Dap**-Arg-Phe-NH_2_	C_58_H_83_N_15_O_11_	anticonvulsant activity, [53]
34	7	Val-Val-Tyr-**Ac5c**-Trp-Thr-**Dab**-Arg-Phe-NH_2_	C_59_H_85_N_15_O_11_	anticonvulsant activity, [53]
35	8	Val-Val-Tyr-**Ac6c**-Trp-Thr-**Dap**-Arg-Phe-NH_2_	C_59_H_85_N_15_O_11_	anticonvulsant activity, [53]
36	9	Val-Val-Tyr-**Ac6c**-Trp-Thr-**Dab**-Arg-Phe-NH_2_	C_60_H_87_N_15_O_11_	anticonvulsant activity, [53]
37	H7-1	**Ile**-Val-Val-Tyr-Pro-Trp-Thr-Gln-Arg-**D-Phe**-NH2	C_65_H_94_N_16_O_13_	anticonvulsant activity, [54]
38	H7-2	**Ile**-Val-Tyr-Pro-Trp-Thr-Gln-Arg-**D-Phe**-NH2	C_60_H_85_N_15_O_12_	anticonvulsant activity, [54]
39	H7-3	**D-Leu**-Val-Val-Tyr-Pro-Trp-Thr-Gln-Arg-**D-Phe**-NH_2_	C_65_H_94_N_16_O_13_	anticonvulsant activity, [54]
40	H7-4	**D-Val**-Val-Tyr-Pro-Trp-Thr-Gln-Arg-**D-Phe**-NH_2_	C_59_H_83_N_15_O_12_	anticonvulsant activity, [54]
41	H7-5	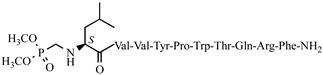	C_68_H_101_N_16_O_16_P	anticonvulsant activity, [54]
42	H7-6	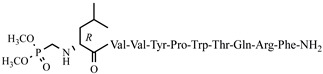	C_68_H_101_N_16_O_16_P	anticonvulsant activity, [54]
43	H7-7	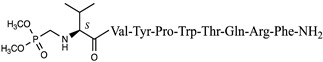	C_62_H_90_N_15_O_15_P	anticonvulsant activity, [54]
44	H7-8	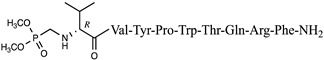	C_62_H_90_N_15_O_15_P	anticonvulsant activity, [54]
45	Dm-7	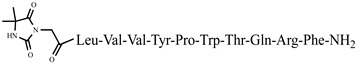	C_72_H_102_N_18_O_16_	anticonvulsant activity, [45]
46	Ph-7	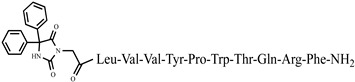	C_82_H_106_N_18_O_16_	anticonvulsant activity, [45]
47	RGD1	Val-Val-Tyr-Pro-Trp-Thr-Gln-Arg-Phe-**Arg-Gly-Asp**-NH_2_	C_71_H_103_N_21_O_17_	antinociceptive activity, [55]
48	RGD2	**Asp-Gly-Arg**-Val-Val-Tyr-Pro-Trp-Thr-Gln-Arg-Phe-**Arg-Gly-Asp**-NH_2_	C_83_H_123_N_27_O_22_	antinociceptive activity, [55]
49	NH7C	**Nic**-Leu-Val-Val-Tyr-Pro-Trp-Thr-Glu-Arg-Phe-**Cys**-NH_2_	C_74_H_101_N_17_O_16_S	antiviral and antibacterial activity, [52]
50	NCH7	**Nic-Cys**-Leu-Val-Val-Tyr-Pro-Trp-Thr-Glu-Arg-Phe-NH_2_	C_74_H_101_N_17_O_16_S	antiviral and antibacterial activity, [52]

**Table 2:** Quantitative assessment of anticonvulsant activity of hemorphin peptides in the MES test in mice.

Drug	TPE ^a^	ED_50_^b^ µg	95% Confidence Interval	TD_50_^c^	PI ^d^
	(min)				
Phenytoin	60	4.92 mg.kg^−1^	(2.57–9.39)	>100 mg.kg^−1^	>20.35
Hemorphin-4 analogs	10				
P4-1		-	-	-	-
P4-2		2.33	(1.13–4.83)	>10	>4.29
P4-3		1.66	(1.24–2.24)	>10	>6.02
P4-4		2.33	(1.13–4.83)	>10	>4.29
P4-5		0.41	(0.19–0.90)	>10	>24.39
Peptide-based chemosensor bearing azobenzene side chain bio photoswitch	10				
Cis Az-H4		1.71	(1.16–2.51)	>10	>5.85
Trans A-H4		1.51	(1.04–2.02)	>10	>6.62
VV-Hemorphin-5 analogs	10				
V2		-	-	-	-
V3		-	-	-	-
V4		3.63	(2.45–5.38)	>20	>5.51
V5		3.19	(2.62–3.87)	>20	>6.27
V6		16.77	(11.08–25.36)	>20	>1.19
V7		16.55	(12.78–21.41)	>20	>1.21
5,5-dimethyl- and 5,5-diphenylhydantoin-conjugated hemorphin derivatives	10				
Dm-4		0.36	(0.13–1.0)	>3	>8.33
Dm-5		0.74	(0.06–8.8)	>5	>6.76
Dm-7		0.7	(0.05–9.58)	>10	>14.29
Ph-4		0.56	(0.06–5.34)	>8	>14.29
Ph-5		0.25	(0.10–0.60)	>5	>20
LVV- and VV-hemorphin-7 analogs	10				
H7-1		-	-	-	-
H7-2		0.94	(0.36–2.47)	>8	>8.51
H7-3		0.68	(0.19–2.51)	>8	>11.76
H7-4		2.54	(1.38–4.64)	>15	>5.91
H7-5		1.53	(0.60–3.88)	>3	>1.96
H7-6		0.38	(0.13–1.15)	>3	>7.89
H7-7		1.58	(0.68–3.70)	>5	>3.16
H7-8		1.67	(1.11–2.51)	>7	>4.19

The values represented are in the 95% confidence interval. ^a^ Time to peak effect—TPE; ^b^ median effective doses (ED_50_); ^c^ median minimal neurotoxic doses (TD_50_); ^d^ protective index (PI) (rotarod TD_50_/ED_50_).

**Table 3:** Quantitative assessment of anticonvulsant activity of hemorphin peptides in the 6 Hz test in mice.

Drug	TPE ^a^	ED_50_^b^	95% Confidence Interval	TD_50_^c^	PI ^d^
	(min)	µg			
Hemorphin-4 analogs	10				
P4-1		0.52	(0.33–0.82)	>5	>9.62
P4-2		2.16	(1.87–2.49)	>5	>2.31
P4-3		0.83	(0.57–1.19)	>5	>6.02
P4-4		0.44	(0.25–0.78)	>5	>11.36
P4-5		0.64	(0.40–1.02)	>5	>7.81
VV-Hemorphin-5 analogs	10				
V2		9.97	(9.07−10.90)	>20	2
V4		5.09	(4.31–6.02)	>20	3.93
V5		9.89	(8.64–11.34)	>20	2.02
V6		5.55	(5.51–5.58)	>20	7.84
V7		6.61	(6.59–6.62)	>20	3.03
N-modified analogs of VV-hemorphin-5 with aminophosphonate moiety	10				
V2p					
V3p		6.47	(3.96–10.57)	>20	3.09
V4p		4.31	(2.76–10.47)	>20	4.64
V5p		12.55	(9.26–16.99)	>30	2.39
V6p		14.11	(9.17 –21.47)	>40	2.83
5,5-dimethyl- and 5,5-diphenylhydantoin-conjugated hemorphin derivatives	10				
Dm-4		0.53	(0.38–0.73)	>5	9.43
Dm-5		0.64	(0.40–1.01)	>5	7.81
Dm-7		0.54	(0.26–1.11)	>5	9.26
Ph-4		0.22	(0.13–0.37)	>5	22.72
Ph-5		0.27	(0.11–0.69)	>5	18.52
Ph-7		0.23	(0.10–0.52)	>5	21.74
VV Hemorphin-7 analogs containing unnatural amino acids	10				
VV-H-2		5.69	(3.67–8.81)	>30	>5.27
VV-H-3		5.69	(3.67–8.81)	>30	>5.27
VV-H-4		3.83	(1.50–9.76)	>20	>5.22
VV-H-5		0.89	(0.54–1.46)	>20	>22.47
VV-H-6		2.67	(4.67–8.19)	>20	>7.49
VV-H-7		0.89	(0.66–2.00)	>20	>22.47
VV-H-8		1.01	(0.29–3.55)	>20	>19.80
VV-H-9		1.09	(0.40–3.00)	>30	>27.52
LVV- and VV-hemorphin-7 analogs	10				
H7-1		0.33	(0.32–0.33)	>5	>15.15
H7-2		2.6	(1.58–4.55)	>5	>1.87
H7-3		-	-	-	-
H7-4		-	-	-	-
H7-5		2.16	(1.70–2.74)	>5	>2.31
H7-6		2.44	(1.41–4.21)	>5	>2.05
H7-7		2.16	(1.70–2.74)	>5	>2.31
H7-8		3.03	(2.44–3.74)	>5	>1.65

The values represented are in the 95% confidence interval. ^a^ Time to peak effect—TPE; ^b^ median effective doses (ED_50_); ^c^ median minimal neurotoxic doses (TD_50_); ^d^ protective index (PI) (rotarod TD_50_/ED_50_).

**Table 4:** Analysis of anticonvulsant activity of hemorphin peptides in *iv*PTZ seizure test in mice.

Drug	TPE ^a^	Comparison of Activity Related to the Threshold Dose (µg/10 µL) for Clonic Seizures
	(min)	
Hemorphin-4 analogs	10	
P4		
P4-2		P4-4 = P4-5 > P4-2 = P4-3 > P4
P4-3		
P4-4		
P4-5		
VV-Hemorphin-5 analogs	10	
V1		
V2		V1 = V4 > V2
V4		
V5		
V6		
V7		
N-modified analogs of VV-hemorphin-5 with aminophosphonate moiety	10	V1 = V3p
V1		
V2p		
V3p		
V4p		
V5p		
V6p		
Hemorphin-7 analogs containing unnatural amino acids	10	VV–H7 = V–H4
VV-H7		
VV-H-2		
VV-H-3		
VV-H-4		
VV-H-5		
VV-H-6		
VV-H-7		
VV-H-8		
VV-H-9		
LVV- and VV-hemorphin-7 analogs	10	
H7		H7-5> H7 = H7-3 = H7-6 = H7-7 = H7-8 > H7-1
H7-1		
H7-2		
H7-3		
H7-4		
H7-5		
H7-6		
H7-7		
H7-8		

The values represented are in the 95% confidence interval. ^a^ Time to peak effect—TPE.

**Table 5:** Bioactive hemorphin peptides.

Drug	Test	Concentration/Dose	Effect	Reference
Endogenous tetrapeptides endomorphin-1 (Tyr-Pro-Trp-Phe-NH_2_), endomorphin-2 (Tyr-Pro-Phe-Phe-NH_2_), morphiceptin (Tyr-Pro-Phe-Pro-NH_2_), hemorphin-4 (Tyr-Pro-Trp-Thr), Tyr-MIF-1 (Tyr-Pro-Leu-Gly-NH_2_), Tyr-W-MIF-1 (Tyr-Pro-Trp-Gly-NH_2_), TAPS (Tyr-D-Arg-Phe-Sar), and DALDA (Tyr-D-Arg-Phe-Lys-NH_2_)	in vitro LC neurons	TAPS IC_50_ = 1.9 nM > endomorphin-1 (IC_50_ = 8.8 nM) and endomorphin-2 (IC_50_ = 5.3 nM) > DALDA IC_50_ = 20 nM) > morphiceptin (IC_50_ = 65 nM) > Tyr-W-MIF-I IC_50_ = 3.8 µM > hemorphin-4 IC_50_ = 6.7 µM > Tyr-MIF-1 IC_50_ = 37.5 µM	inhibition of the spontaneous firing	[17]
VV-Hemorphin-5 Valorphin (endogenous Hb β-chain (33–39) fragment) Valorphin Valorphin Valorphin	hot plate, tail flick test in mice; Randall–Selitto test in rat in vitro cerebellar Purkinje cells the guinea pig ileum muscle preparation L929 and K562 tumor cells tumor (L929 and A549) cell cultures, primary culture of murine bone marrow cells and in murine model of breast carcinoma in vivo	IC_50_ of 14 nM IC_50_ of 10 µM 10^−7^–10^−13^ M concentration range +1 µM	opioid analgesic activity binds to rat mu-opioid receptor inhibition of the spontaneous firinginhibition of the electrically induced contractions of mu-opioid receptor Tumor cell cytolysis additive effects 0.5 µM epirubicin added 24 h prior to 1 µM valorphin; 1 µM valorphin added 48 h prior to 0.1 µM epirubicin - 100% cell death	[23] [24] [26] [27]
Hemorphin-6 and Hemorphin-7 LVV-hemorphin-7 LVV-hemorphin-7 LVV-hemorphin-7 and alanine-containing derivates of Lev-Val-Val-Hemorphin-7 LVV-hemorphin-6 LVV-hemorphin-7 LVV-hemorphin-7	(in vitro) distal end of the sciatic nerve radiotelemetry (blood pressure) in WK rats and SHRs Tail-flick and hot plate tests in chronic alcohol-exposed rat model, ELISA Rat blood pressure assay OF, EPM, FST tests in rat carrageenan-induced hyperalgesia at the spinal level Barnes circular maze in rat	20 and 200 µM 100 µg/kg - ED25 128.0 (nmoles/kg) 153 nmol/kg i.p. 27.2 nmol i.t. 100 pmol	electrical stimulation Reduce blood pressure Decreased plasma level of LVV-hemorphin-7 pressor and tachycardic activities mediated by the SNS anxiolytic and antidepressant effect decrease enhanced spatial learning	[74] [29] [34] [32] [75] [76] [77]

The values represented are in the 95% confidence interval.

**Table 6:** Parameters of the voltammetric measurements and the electrochemical data of peptides.

Peptide	pKa_1;_ pKa_2_ Constants	
Hemorphin-4 Analogs	Method Determination	Constants, Reference
P4	by potentiometric titration	3.80; 6.44, [44]
P4-1		3.89; 6.52, [44]
P4-2		3.93; 6.71, [44]
P4-3		3.88; 6.93, [44]
P4-4		6.16; 8.90, [44]
P4-5		6.20; 9.06, [44]
Dm-4	by potentiometric titration	2.86; [45]
Ph-4	by potentiometric titration	2.98; [45]
Rh-1	by potentiometric titration	2.81; 6.60, [47]
Rh-2		2.78;6.38, [47]
Rh-3		2.86;6.39, [47]
Hemorphin-5 analogs		
V2/H2	by potentiometric titration	9.23, [48,49]
V3/H3		8.12, [48,49]
V4/H4		7.83, [48,49]
V5/H5		8.24, [48,49]
V6/H6		8.01, [48,49]
V7/H7		8.17, [48,49]
V2p	by potentiometric titration and voltamperometry	8.93, [50,51]
V3p		8.83, [50,51]
V4p		7.92, [50,51]
V5p		8.97, [50,51]
V6p		9.05, [50,51]
Dm-5	by potentiometric titration	3.06; 7.14, [45]
Ph-5		3.09; 6.98, [45]
C-V	by fluorimetry	5.18, [52]
H-V		4.75, [52]
AC-V		5.43, [52]
AH-V		4.84, [52]
Hemorphin-7 analogs		
2	by potentiometric titration	8.04(Val); 5.34(Tyr), [53]
3		7.49(Val); 4.83(Tyr), [53]
4		7.10(Val);5.46(Dap, Dab); 3.14(Tyr), [53]
5		8.08(Val);7.21(Dap, Dab); 5.98(Tyr), [53]
6		8.15(Val);6.87(Dap, Dab); 4.72(Tyr), [53]
7		8.21(Val);7.26(Dap, Dab); 4.70(Tyr), [53]
8		9.20(Val);8.03(Dap, Dab); 5.27(Tyr), [53]
9		9.08(Val);8.80(Dap, Dab); 4.66(Tyr), [53]
H7-1	by potentiometric titration	2.98; 6.12, [54]
H7-2		3.09; 6.62, [54]
H7-3		3.05; 6.78, [54]
H7-4		3.22; 6.52, [54]
H7-5		3.17; 6.23, [54]
H7-6		2.98; 5.85, [54]
H7-7		3.15; 6.09, [54]
H7-8		2.78; 5.52, [54]
Dm-7	by potentiometric titration	3.19; 5.11, [45]
Ph-7		3.23; 6.45, [45]
RGD1	by potentiometric titration	3.53; 6.42, [55]
RGD2		3.48; 6.34, [55]
NH7C	by fluorimetry	5.07, [52]
NCH7		4.78, [52]

**Table 7:** Parameters of the voltammetric measurements and the electrochemical data of peptides.

Peptide	Working Electrode/Electrolyte		E_p,a_ [V]	E_p,c_ [V]	Nature of the Process, Reference
Hemorphin-4 Analogs	Electrod	Electrolyte			
Dm-4	Hg(HMDE)	Methanol/ tetrabutylamonium persulfat (0.043 mol L^−1^)	0.050	-	IR [45]
Ph-4	Hg(HMDE)		E_p1_ = 0.116 E_p2_ = −0.181 E_p3_ = −0.657	- E_p2_ = −0.171 E_p3_ = −0.518	R [45]
Az-H4	Hg(HMDE)	(1) pH 6.86 (phosphate-buffered solution, 0.1 mol L^−1^)(2) AcCN	(1) −0.547 (2) −0.603	(1) 0.458 (2) 0.355	(1) R * [46] (2) R [46]
Hemorphin-5 analogs					
V2/H2	Pt-working electrode SW	phosphates buffer at pH 6.86,	0.385 0.217	- −0.106	QR [48,49]
V3/H3			0.355 0.156	- −0.175	QR [48,49]
V4/H4			0.400 0.171	- −0.045	QR [48,49]
V5/H5			0.370 0.187	- −0.060	QR [48,49]
V6/H6			0.370 0.170	- −0.045	QR [48,49]
V7/H7			0.370 0.171	- −0.075	QR [48,49]
V2p	glass carbonic (GC) electrode SW	Phosphate-buffered solution (pH 6.86)	−0.708 −0.0476 0.315 0.661	0.00488 −0.465 −0.751	[50,51]
V3p			−0.708 −0.0476 0.315 −0.661	0.00488 −0.465 −0.751	[50,51]
V4p			−0.661 0.143 0.411	−0.013 0.727 -	[50,51]
V5p			−0.576 0.638	−0.0534 −0.708	[50,51]
V6p			−0.436 0.254 0.526	0.144 −0.732 -	[50,51]
Dm-5	Hg(HMDE)	Methanol/ tetrabutylamonium persulfat (0.043 mol L^−1^)	E_p1_ = 0.105 E_p1_ = −0.252 **	E_p1_ = 0.270	QR[45]
Ph-5	Hg(HMDE)		E_p1_ = 0.186 E_p1_ = −0.439 **	E_p1_ = 374	QR[45]
C-V	glass-carbon (GC)	phosphate-buffered solution at (1) pH 6.87 and (2) 7.34 DPP	(1) −0.625(Trp) −1.26(Tyr) (2) −0.625(Trp) −1.26(Tyr) 1.82(Cys)	-	IR [52]
H-V			(1) −0.661(Trp) −1.297(Tyr) (2) −0.661(Trp) −1.297(Tyr) 1.82(His)	-	IR [52]
AC-V			(1) −0.619(Trp) −1.19(Tyr) (2) −0.619(Trp) −1.19(Tyr) 1.75 (Cys)	-	IR [52]
AH-V			(1) −0.631(Trp) −1.270(tyr) (2) −0.631(Trp) −1.270(tyr) 1.78(His)	-	IR [52]
Hemorphin-7 analogs					
2	(1) HMDE electrode (SW) (2) Au electrode (SW)	LiOH/LiCl, pH = 10.65	(1) −1.70 (2) 0.179 1.27	(1) −1.74 (2) −0.0563 0.501	(1) R [53]
3			(1) −1.74 (2) 0.179 1.27	(1) −1.80 (2) −0.0563 0.501	(1) R [53]
4			(1) −1.72 (2) 0.179	(1) −1.80 (2) −0.0592	(1) R [53]
5			(1) −1.73 (2) 0.179	(1) −1.78 (2) −0.0592	(1) R [53]
6			(1) −1.71 (2) 0.160	(1) −1.76 (2) −0.0560 −0.219 0.341	(1) R [53]
7			(1) −1.66 (2) −0.0465 0.569 0.733 1.27	(1) −1.70 (2) 0.453 0.304 −0.220 −0.0560	(1) R [53]
8			(1) −1.75 (2) 0.179	(1) −1.76 (2) −0.0392 0.441	(1) R [53]
9			(1) −1.73 (2) 0.179	(1) −1.78 (2) −0.0592 0.461	(1) R [53]
H7-1	glass carbonic (GC) electrode DPP	phosphate-buffered solution at pH 7.04	0.762	-	IR [54]
H7-2			0.762	-	IR [54]
H7-3			0.756	-	IR [54]
H7-4			0.696	-	IR [54]
H7-5			0.756	-	IR [54]
H7-6			0.756	-	IR [54]
H7-7			0.756	-	IR [54]
H7-8			0.756	-	IR [54]
Dm-7	Hg(HMDE)	Methanol/tetrabutylamonium persulfat (0.043 mol L^−1^)	E_p1_ = 0.093 E_p2_ = −0.608	- E_p2_ = −0.470	IR [45]
Ph-7	Hg(HMDE)		E_p1_ = 0.106 E_p2_ = −0.618	- E_p1_ = −607	R [45]
RGD1	Hg(HMDE) (CV)	Methanol/tetrabutylamonium persulfat (0.043 mol L^-1^)	−0.44	−0.54	R[55]
RGD2	Hg(HMDE) (CV)		−0.460	−0.560	R[55]
NH7C	glass-carbon (GC) DPP	phosphate-buffered solution at (1) pH 6.87 and (2) 7.34	(1) −0.518(Trp) −1.196(Tyr) (2) −0.518(Trp) −1.196(Tyr)	-	(1) QR [52]
NCH7			(1) −0.613(Trp) −1.17(Tyr) (2) −0.613(Trp) −1.17(Tyr)	-	(1) QR [52]

* R—reversible; QR—quasireversible; IR—irreversible; ** at high concentrations.

## Data Availability

Data sharing not applicable.
